# Atypical Protein Kinase C Zeta: Potential Player in Cell Survival and Cell Migration of Ovarian Cancer

**DOI:** 10.1371/journal.pone.0123528

**Published:** 2015-04-14

**Authors:** Kelly K. Y. Seto, Irene L. Andrulis

**Affiliations:** 1 Department of Molecular Genetics, University of Toronto, Toronto, Ontario, Canada; 2 Lunenfeld-Tanenbaum Research Institute, Mount Sinai Hospital, Toronto, Ontario, Canada; The University of Hong Kong, HONG KONG

## Abstract

Ovarian cancer is one of the most aggressive gynaecological cancers, thus understanding the different biological pathways involved in ovarian cancer progression is important in identifying potential therapeutic targets for the disease. The aim of this study was to investigate the potential roles of Protein Kinase C Zeta (PRKCZ) in ovarian cancer. The atypical protein kinase C isoform, PRKCZ, is involved in the control of various signalling processes including cell proliferation, cell survival, and cell motility, all of which are important for cancer development and progression. Herein, we observe a significant increase in cell survival upon PRKCZ over-expression in SKOV3 ovarian cancer cells; additionally, when the cells are treated with small interference RNA (siRNA) targeting *PRKCZ*, the motility of SKOV3 cells decreased. Furthermore, we demonstrate that over-expression of PRKCZ results in gene and/or protein expression alterations of insulin-like growth factor 1 receptor (IGF1R) and integrin beta 3 (ITGB3) in SKOV3 and OVCAR3 cells. Collectively, our study describes PRKCZ as a potential regulatory component of the IGF1R and ITGB3 pathways and suggests that it may play critical roles in ovarian tumourigenesis.

## Introduction

*PRKCZ* encodes a protein belonging to the atypical subclass of the protein kinase C family of serine/threonine kinases that has been implicated in the regulation of cellular transformation and carcinogenesis [1]. PRKCZ has previously been observed to be involved in multiple signal transduction pathways, including activation of the ERK/MAPK cascade, p70 ribosomal S6 kinase signalling cascade, transcription factor NF-κB, as well as regulation of cell polarity [2]. The regulation of these pathways may explain some of the mechanisms by which PRKCZ can promote human cancers. Indeed, the roles of PRKCZ in various cancer types have been examined in recent years. For example, it was reported that *PRKCZ* expression level is two fold higher in glioblastoma cell lines compared with normal astrocytes [3]. Subsequent studies showed that this high level of expression is correlated with increased proliferation of glioblastoma cells, while reduced expression is correlated with inhibition of migration and invasion [3,4,5]. The involvement of activated PRKCZ in epidermal growth factor (EGF)-induced chemotaxis has also been examined in lung and breast cancer, and it was shown that PRKCZ is able to elicit a migration response of these cells by acting as a downstream mediator in the phosphatidylinositol 3-kinase (PI3K)/AKT pathway [6,7]. Additionally, PRKCZ participates in cell polarity pathways, and studies have illustrated that loss of cell polarity, which results in tissue disorganization, may contribute to cancer development [8]. It has also been observed that PRKCZ is mislocalized in a subset of ovarian cancers, and it was suggested that this mislocalization may reflect a role for apical-basal loosening, thus disrupting cell-cell adhesion, as well as increasing cell growth [9]; however, additional evidence supporting the role of PRKCZ in ovarian cancer remains limited.

In the present study, we tested the hypothesis that PRKCZ plays a role in ovarian cancer cell viability, proliferation and migration. We detected an increase in cell proliferation in SKOV3 cells when PRKCZ was over-expressed. Moreover, SKOV3 cells exhibited a decrease in cell migration when endogenous PRKCZ expression was down-regulated by small-interference RNA (siRNA). Our data further illustrate that up-regulation of PRKCZ leads to expression alterations of IGF1R and ITGB3 in SKOV3 and OVCAR3 cell lines, suggesting that PRKCZ may participate in ovarian cancer progression by modulating the expression of other important signalling molecules.

## Materials and Methods

### Cell Culture

Ovarian cancer cell lines SKOV3 and OVCAR3 were purchased from American Type Culture Collection (Manassas, VA). SKOV3 cells were maintained in McCoy’s medium supplemented with 10% FBS. OVCAR3 cells were maintained in RPMI-1640 medium supplemented with 20% FBS and 0.01 mg/ml bovine insulin. Cells were incubated at 37°C in a humidified atmosphere of 5% CO_2_ and 95% air.

### *PRKCZ* Expression Vector & Generation of Stable Clones

PCR conditions to amplify human *PRKCZ* in a 25 μL reaction volume were as follows: 2.5 μL of 10X Platinum HiFidelity Buffer (Invitrogen), 1.5 μL of 10 mM dNTPs (Invitrogen), 1.0 μL of 50 mM MgSO_4_ (Invitrogen), 0.3 μL of 30 μM EcoRI-tagged forward primer (5’-TCATGAATTCACTATGCCCAGCAGGACC-3’), 0.3 μL of 30 μM SalI-tagged reverse primer (5’-CATAGTCGACACACCGACTCCTCGGT-3’), 0.5 μL of Platinum HiFidelity *Taq* Polymerase (5U/μL, Invitrogen), 17.9 μL of ddH_2_O, and 1 μL (50 ng) of pooled human cDNA (derived from 13 human cell lines: NTERA-2, Hs578T, HepG2, Ht1080, SW872, T45D, MCF-12A, SKOV3, Fetal Normal Muscle Cells, Colo-205, MOLT-4, RPMI 8226, and SK-MEL-28). Thermal cycling parameters were as follows: initial incubation for 2 minutes at 94°C; 40 cycles of 30 seconds at 94°C, 30 seconds at 73°C, 2 minutes at 72°C. PCR products were resolved by 1.0% agarose gel electrophoresis, visualized under UV, and gel extracted and purified according to the manufacturer’s protocol (Qiagen). Subsequently, they were transferred to pEGFP-N2 (N-terminal GFP tag) expression vector (Clontech). Correct *PRKCZ* sequence within vector was confirmed by sequencing. Each cell line was transfected with the plasmid vectors PRKCZ-pEGFP or vector controls, using Fugene 6 Transfection Reagent (Roche). Following transfection, cells were cultured with G418 sulfate (800 μg/ml and 500 μg/ml for SKOV3 and OVCAR3, respectively). Surviving colonies were individually selected and maintained in G418 sulfate-containing medium.

### Quantitative Real-Time PCR

Primer pairs for genes of interest were designed individually by using Primer3 input software (Whitehead Institute, Howard Hughes Medical Institute, NIH). *PRKCZ*-forward: 5’-GGCCACAGACTGGATTTTCT-3’, *PRKCZ*-reverse: 5’- CTCGCTGGTGAACTGTGTGT-3’; *IGF1R*-forward: 5’-GGTGGAGAACGACCATATCC-3’, *IGF1R*-reverse: 5’-GCCAGCGCACAATGTAGTAA-3’; *ITGB3*-forward: 5’-ATGGGACACAGCCAACAACC-3’, *ITGB3*-reverse: 5’-GTGGCACAGGCTGATAATGA-3’; *TIMP1*-forward: 5’-TGACCAAGATGTATAAAGGG-3’, *TIMP1*-reverse: 5’-GTTGTGGGACCTGTGGAAGT-3’. Quantitative real-time RT-PCR was performed on an ABI Prism 7000 Sequence Detector (Applied Biosystems) using SYBR Green PCR Master Mix (Applied Biosystems). Each of the 20 μL PCR reactions contained 1 μL (50 ng) of cDNA and 0.45 μM of each of the primers. The thermal cycles for PCR reaction were as follow: initial denaturation for 10 minutes at 95°C, followed by 40 cycles of 95°C for 15 seconds, and annealing extension at 60°C for 1 minute. The housekeeping gene *HPRT1* (forward: 5’- ATGGTCAAGGTCGCAAGCTTG-3’, reverse: 5’- CAAATCCAACAAAGTCTGGCT-3’) was used to normalize gene expression values. Reference cDNA consisted of a pool of 13 cell lines was used to generate standard curve to quantify cDNA levels of samples.

### Western Blotting

Cells were washed three times with cold phosphate-buffered saline (PBS), lysed with NETN lysis buffer (20mM Tris-HCl, pH 7.5; 150mM NaCl; 1 mM EDTA, pH 8.0; 0.5% Nonidet P-40; 1 mM PMSF; 1x protease and phosphatase inhibitors) on ice for 10 minutes and centrifuged for 8 minutes at 12,000 rpm to separate lysates from cell debris. Protein concentrations were determined with BCA Protein Assay Kit (Pierce). Equal amounts of protein from cell lines were loaded and separated on 8–10% SDS-PAGE. Proteins were transferred to Hybond ECL nitrocellulose (Amersham) and blotted using anti-PRKCZ, anti-IGF1R, anti-ITGB3, anti-IRS2 (Cell Signalling), and anti-pIRS2 (Abcam) antibodies at 1:1000 dilutions. Secondary conjugates, HRP-Donkey anti-mouse or HRP-Donkey anti-rabbit (Jacksons Immunochemicals) were incubated for 1 hour at a 1:5000 dilution. Protein bands were visualized by chemiluminescence using ECL detection system (Amersham).

### Cell Viability Assays

In brief, cells were seeded in 96-well plates at a concentration of 1000 cells/well with a final volume of 100 μL of culture media and were incubated at 37°C, with or without myristoylated pseudosubstrate peptide (40 μM), a PRKCZ inhibitor. After each incubation period, 10 μL of the MTT (3-(4,5-Dimethylthiazol-2-yl)-2,5-diphenyltetrazolium bromide) labelling reagent (Roche) were added to each well at a final concentration of 0.5 mg/mL. Cells were incubated for an additional 4 hour period, followed by addition of 100 μL of solubilization solution (10% SDS in 0.01 M HCl). Plates were allowed to stand overnight at 37°C and the spectrophotometrical absorbance of the samples was measured using a microplate (ELISA) reader at a wavelength of 570 nm with background subtraction at 630 nm. Each assay was performed in triplicates.

### BrdU Proliferation Assay

Approximately 1 x 10^5^ cells were plated onto coverslips within 6-well plates and allowed to grow to ~60% confluency overnight. On the next day, cells were incubated in 10uM of BrdU for 6 hours, washed with PBS, then fixed with 4% paraformaldehyde, denatured with 2M HCl, and neutralized with 0.1 M sodium borate. Cells were then incubated with mouse anti-BrdU (1:200, Dako) for 1 hour, washed, followed by incubation with Alexa Fluro 647 anti-mouse (Molecular Probes) for an additional 30 minutes, and counterstained with DAPI. BrdU incorporation was observed and counted in five fields of view per well through microscopy. Each cell line was performed in triplicates per experiment and the ratio of BrdU-positive cells to total cell number was calculated.

### Scratch Wound Healing Assay

Cells were plated into 6-well plates and cultured to confluence. Cells were rinsed with PBS and serum starved overnight in 0.5% FBS media at 37°C and 5% CO_2_. The next day, three separate scratches were introduced through the monolayer of cells in each of the wells using sterile 200 μL plastic pipette tips. Cells were then rinsed gently with PBS to remove cellular debris and replaced with fresh culture media supplemented with 0.5% FBS. The wounded cells were allowed to incubate at 37°C and representative fields were photographed with an inverted-phase microscope at different time intervals.

### siRNA Transfections

Knockdown of *PRKCZ*, *IGF1R*, and *ITGB3* expression in ovarian cancer cell lines was achieved by transfection of siRNAs (Ambion). siRNAs targeting of these genes was performed with Dharmafect-4 transfection reagent (Dharmacon). In brief, cells were seeded in 12-well or 6-well plates at densities of 1 x 10^5^ or 2 x 10^5^ cells/well, respectively. Cells were then treated with siRNA transfection mixtures following the manufacturer’s protocol. Scrambled siRNA (Ambion) was used as a control. Additional controls included mock-treated cells that received transfection reagent without siRNA, as well as untreated cells that received only fresh media. Cells were harvested after 48 or 72 hours for RNA and protein extraction, respectively.

### Statistical Analyses

All data were represented as means ± the standard deviation (SD) of the mean. Statistical calculations were performed with Microsoft Excel analysis tools. Differences between groups were analyzed by student *t*-test. *P* values of < 0.05 were considered statistically significant.

## Results

### PRKCZ plays a role in cell viability in SKOV3 ovarian cancer cells

**Fig 1:**
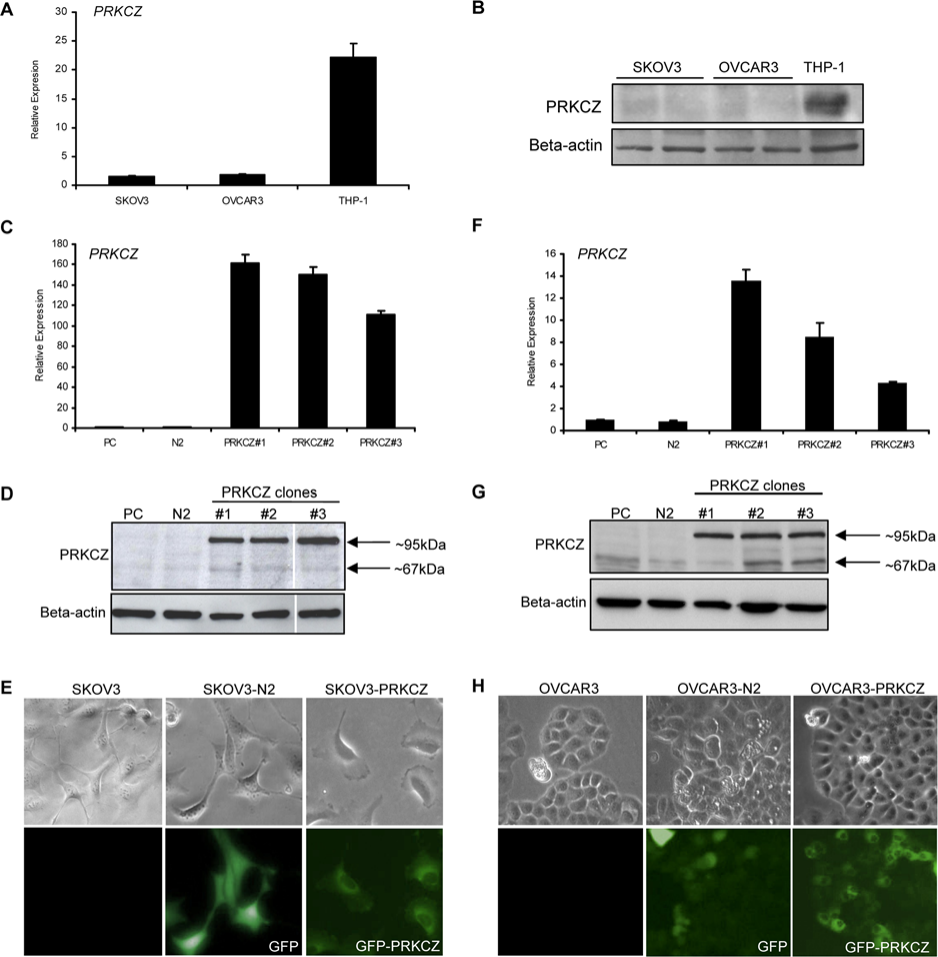
Transcript and protein levels of PRKCZ in parental and *PRKCZ*-transfected SKOV3 and OVCAR cell lines. (A, B) The endogenous transcript and protein levels of PRKCZ are low in both SKOV3 and OVCAR3 cells, as determined by real-time PCR and western blot analysis. Human acute monocytic leukemia cell line THP-1, which expresses a high level of PRKCZ, was used as a positive control. Beta-actin protein was probed on the blot as a loading control. The beta-actin loading control is also shown (lower panel). (C-E) Comparison of PRKCZ transcript and protein expression in SKOV3 parental control (PC), control vector (N2), and a selection of PRKCZ clones by quantitative real-time RT-PCR, western blot analysis with PRKCZ-antibody, and fluorescence microscopy. (F-H) Comparison of PRKCZ transcript and protein expression in OVCAR3 parental cells (PC), control vector (N2), and a selection of PRKCZ clones by quantitative real-time RT-PCR, western blot analyses with PRKCZ-antibody, and fluorescence microscopy. For both SKOV3 and OVCAR3, a band of ~95 kDa was detected by western blotting in stable cell lines that over-express PRKCZ, which corresponded with molecular weight of GFP-tagged PRKCZ protein. A faint band of ~67 kDa corresponded to endogenous PRKCZ. The three PRKCZ clones for both SKOV3 and OVCAR3 were selected for further experiments.

To address the potential roles of PRKCZ in ovarian tumourigenesis, including cell viability, proliferation, cell migration, as well as relevant downstream signalling pathways, we performed several *in vitro* functional assays using ovarian cancer cells. The endogenous PRKCZ transcript and protein levels were low for both SKOV3 and OVCAR3 compared to THP-1 cells, a human acute monocytic leukemia cell line that expresses high endogenous levels of PRKCZ (Fig 1A and 1B).

**Fig 2:**
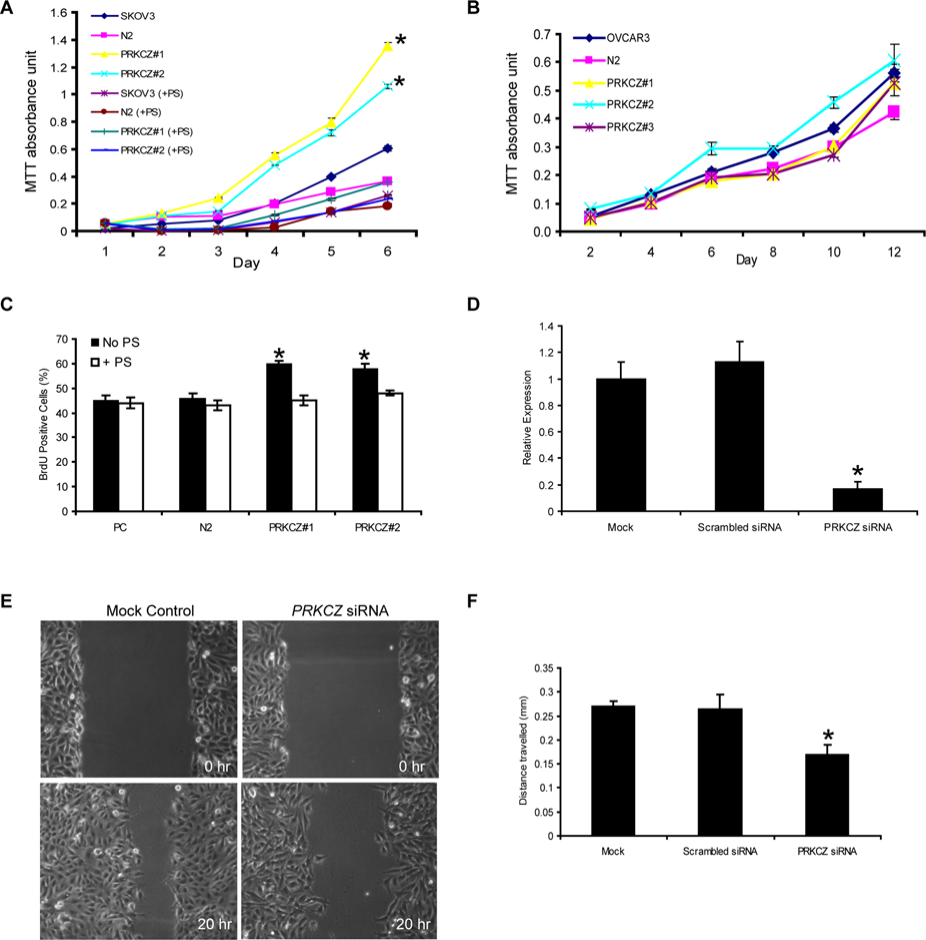
PRKCZ plays roles in cell viability, proliferation and migration in SKOV3 cells. (A) Increased cell viability was observed in cells over-expressing PRKCZ, as measured by MTT cell viability assay. This effect was abolished by the addition of PRKCZ myristoylated pseudosubstrate (PS) (p*<0.05, n = 3). (B) No significant change in cell viability was observed in OVCAR3 cells. (C) BrdU incorporation assay was performed to measure prolifartion rate of SKOV3 cells. PRKCZ-transfected cells showed an increase in BrdU-positive cells, indicating increased cell growth compared to controls (*p<0.01). This effect was reversed by the addition of PRKCZ myristyolated pseudosubstrate (PS). PC = parental control; N2 = empty vector control N2. *PRKCZ* gene knockdown in SKOV3 parental cells affects migration as observed by wound healing assay. (D) Confirmation of *PRKCZ* gene expression knockdown by quantitative real-time PCR (*p<0.01). (E, F) Knockdown of *PRKCZ* expression by siRNA inhibits migration compared to controls as observed in wound healing assays (*p<0.01; n = 3).

PRKCZ has previously been demonstrated to be involved in cell survival in various cell types [10,11,12,13,14]. To assess whether it has the same effect on ovarian cancer cells, stable cell lines over-expressing PRKCZ were generated (Fig 1C–1H), and MTT cell viability assays were performed using randomly selected clones. It was observed that over-expression of PRKCZ significantly correlated with an increase in the viability of SKOV3 cells, and this effect was abolished by the addition of a myristoylated pseudosubstrate peptide that targets PRKCZ (Fig 2A). In contrast, over-expression of PRKCZ did not have an effect on OVCAR cell lines when compared to parental and empty-vector control cells (Fig 2B). This result suggests that PRKCZ can enhance cell viability in a subset of ovarian cancer cells.

To investigate whether the increased cell viability seen in SKOV3 cells that over-express PRKCZ was due to an increase in cell proliferation, BrdU cell proliferation assays were performed. SKOV3 cells over-expressing PRKCZ displayed a higher percentage of cells with BrdU incorporation (Fig 2C), indicating that the rate of proliferation in these cells is higher than parental and empty vector controls.

### Knockdown of PRKCZ inhibits the cell migration of SKOV3 ovarian cancer cells

The ability of cancer cells to migrate is one of the key processes in cancer progression. To determine whether PRKCZ can affect the migratory properties of ovarian cancer cells, scratch wound healing migration assays were performed. No migration differences were observed when PRKCZ-transfected cells were compared with controls (data not shown). To examine if the endogenous levels of PRKCZ in parental cells are sufficient for their migration, wound healing assays were repeated with the same ovarian cell lines that have been subjected to *PRKCZ* siRNA knockdown (Fig 2D). It was observed that knocking down the endogenous level of *PRKCZ* can in fact decrease the migration rate of SKOV3 cells (Fig 2E and 2F). This phenotype was not observed in OVCAR3 cells (data not shown).

### IGF1R and ITGB3 are Potential Downstream Effectors of PRKCZ

IGF1R and ITGB3 participate in important cellular signaling pathways involved in cancer development and both have previously been implicated as prognostic factors in ovarian cancer [15,16,17,18]; since PRKCZ plays a role in the regulation of various cellular signal pathways [2], we sought to determine whether PRKCZ can affect the regulation of IGF1R and ITGB3 in ovarian cancer.

**Fig 3:**
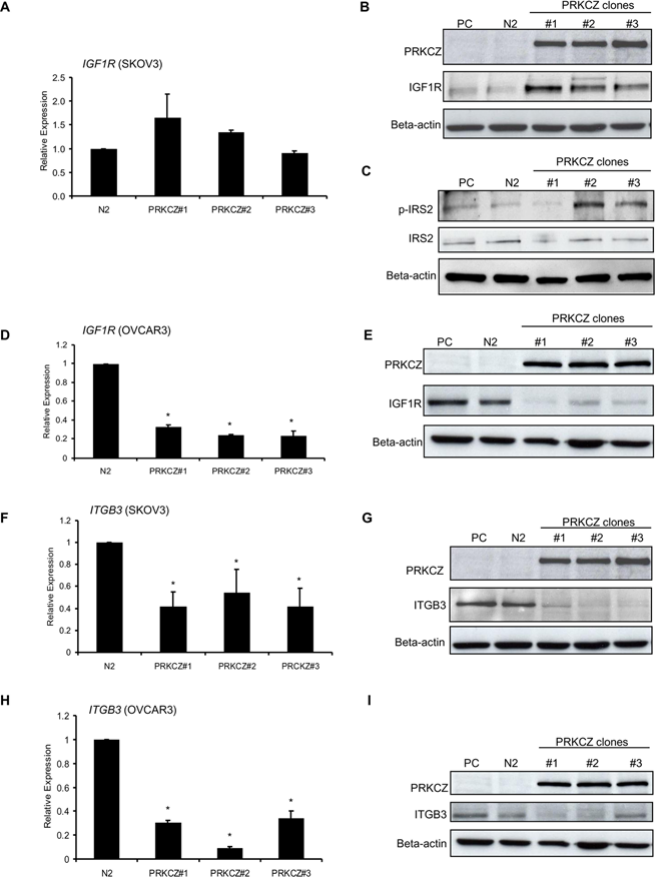
Transcript and protein expression of IGF1R and ITGB3 in parental and PRKCZ-expressing SKOV3 and OVCAR3 cells. (A) No significant increase in *IGF1R* transcript level was observed in PRKCZ-expressing SKOV3 cells. (B) Protein level of IGF1R is elevated in PRKCZ-expressing cells. (C) The level of p-IRS-2, a downstream effector of IGF1R, was elevated in 2 of the 3 PRKCZ clones. (D, E) Both transcript and protein levels of *IGF1R* are decreased in PRKCZ-expressing OVCAR3 cells. This observation is in contrast to SKOV3 cells, which showed increased IGF1R expression at protein levels. (F, G) Both transcript and protein levels of ITGB3 are decreased in PRKCZ-expressing SKOV3 cells. (H, I) Similar to SKOV3 cell line, both transcript and protein levels of *ITGB3* in OVCAR3 cell line are decreased in PRKCZ-expressing cells. PC = parental control; N2 = empty vector control N2. Western blot shown is representative of 3 independent analyses.

To examine if there is a direct molecular relationship between PRKCZ, IGF1R and ITGB3, the expressions of IGF1R and ITGB3 in parental and PRKCZ-expressing ovarian cancer cells were compared at the transcriptional and protein levels. Whereas the transcript level of *IGF1R* was not altered in SKOV3 cells, an increase in PRKCZ protein expression correlated with an increased level of IGF1R protein, suggesting that PRKCZ may participate in IGF1R translation or protein stability (Fig 3A and 3B). Additionally, the expression level of phosphorylated IRS-2 (insulin-receptor substrate-2), a known downstream target for IGF-IR, was also increased in SKOV3 cells (Fig 3C), confirming that PRKCZ is involved in the activation of IGF1R signalling pathway in this particular cell line. Interestingly, in contrast to SKOV3 cells, expression of *IGF1R* was decreased in OVCAR3 cells that over-express PRKCZ, at both the transcript and protein levels (Fig 3D and 3E).

In addition to IGF1R, mRNA and protein expression of ITGB3 were significantly decreased in both SKOV3 cells and OVCAR3 cells that over-express PRKCZ, compared to parental and empty-vector controls (Fig 3F–3I).

**Fig 4:**
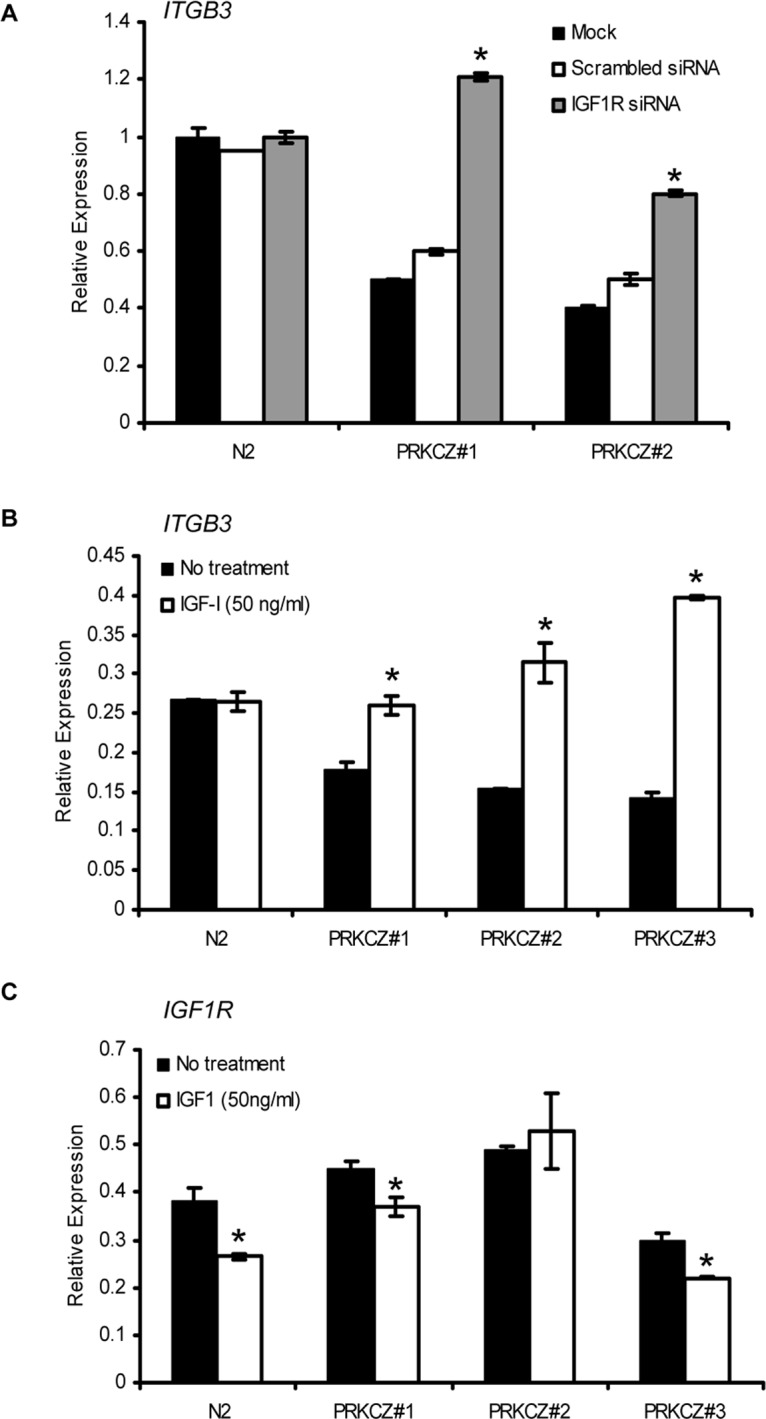
Interplay of gene expression regulation between *PRKCZ*, *IGF1R*, and *ITGB3*. (A) Knockdown of *IGF1R* rescues transcript expression of *ITGB3* in PRKCZ-expressing cells. The expression of *ITGB3* is derepressed in PRKCZ-expressing SKOV3 cells subjected to *IGF1R* gene knockdown by siRNA (*p < 0.05), but *IGF1R* knockdown has no effect on empty vector control. To examine if transcript expression of *ITGB3* is directly regulated by IGF1-signalling, SKOV3 cells were treated with IGF1 (50 ng/ml). (B) The addition of IGF1 had no effect on empty vector control but significantly increased the transcript expression of *ITGB3* in the PRKCZ-expressing clones when compared to their non-treated counterpart (*p < 0.05). (C) When SKOV3 cells are induced with IGF1, the transcript level of *IGF1R* is significantly decreased for two of the three clones (*p<0.05) (C). N2 = empty vector control N2.

The concurrent expression alterations observed for IGF1R and ITGB3 in PRKCZ-expressing cells prompted the question of whether these changes are occurring within the same biological pathway. Therefore, we performed *IGF1R* siRNA gene knockdown experiments in SKOV3 cells to determine if reducing *IGF1R* expression would have an effect on *ITGB3* gene expression. Results from quantitative real-time PCR analysis indicated that *IGF1R* knockdown can lead to derepression of *ITGB3* mRNA expression in cells over-expressing PRKCZ (Fig 4A). To further address whether activation of the IGF1-signalling pathway plays a role in *ITGB3* gene regulation, the transcript level of *ITGB3* was examined after SKOV3 cells were stimulated with IGF1, a known ligand for IGF1R. Interestingly, similar to the *IGF1R* siRNA knockdown experiment, *ITGB3* expression was derepressed in PRKCZ-expressing cells when stimulated with IGF1 (Fig 4B). Moreover, to examine if there is a negative feedback mechanism that regulates expression of *IGF1R*, we induced SKOV3 cells with IGF1 and examined their *IGF1R* mRNA expression (Fig 4C). Upon stimulation with IGF1, the transcript expression of *IGF1R* was decreased in all SKOV3 cells, as observed by quantitative RT-PCR. This suggests that IGF1 stimulation in SKOV3 cells may in fact be able to repress the transcript expression of *IGF1R*.

### TIMP-1 as a Potential Downstream Effector in IGF1 Signalling

**Fig 5:**
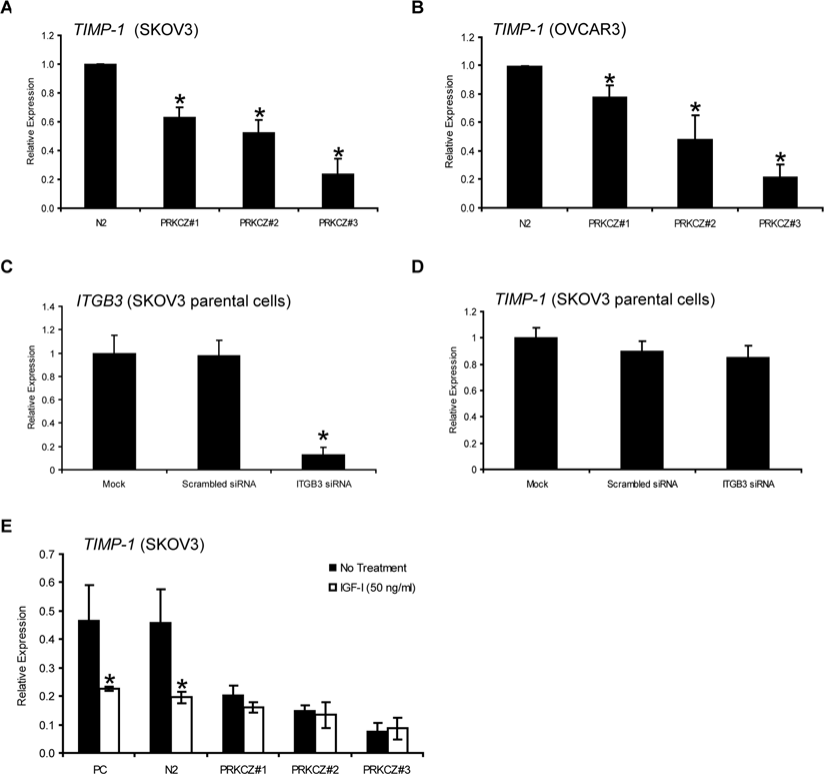
*TIMP1* gene expression regulation in PRKCZ-expressing ovarian cancer cells. (A, B) The mRNA expression of *TIMP1*, a potential downstream target of ITGB3, is decreased in PRKCZ-expressing SKOV3 cells and OVCAR3 cells, as determined by quantitative real-time RT-PCR (*p* < 0.05, n = 3). N2 = empty vector control N2. The transcript levels of *TIMP1*, a potential downstream target of ITGB3, was determined by quantitative real-time RT-PCR following *ITGB3* siRNA knockdown in SKOV3 parental cells. (C) Confirmation of *ITGB3* expression knockdown (*p<0.01). (D) Transcript levels of *TIMP1* in SKOV3 cells with and without *ITGB3* siRNA treatment. No significant *TIMP1* transcript level difference was observed between *ITGB3* siRNA treated and control cells (n = 3). (E) Treatment with IGF1 (50 ng/ml) decreased *TIMP1* transcript levels in SKOV3 parental and empty-vector control cells but not PRKCZ clones (* *p* < 0.05). (n = 2) PC = parental control; N2 = empty vector control N2.

Based on our observations that both ITGB3 mRNA and protein levels are decreased in PRKCZ-expressing cells in two ovarian cancer cell lines (SKOV3 and OVCAR3), we sought to identify potential downstream players within this signalling pathway that may play role in ovarian cancer. Transcription of *TIMP1* (TIMP metallopeptidase inhibitor 1) has previously been shown to be up-regulated by ITGB3 in the ovarian cancer cell line MDAH 2774 and thus may be a candidate target [19]. Therefore, the mRNA level of *TIMP1* in SKOV3 and OVCAR3 cells was examined. Interestingly, *TIMP1* expression was decreased in PRKCZ clones of both of these cell lines, which correlated with the expression of *ITGB3* (Fig 5A and 5B). To further examine if the decrease in *TIMP1* expression was directly related to the decreased level of *ITGB3*, SKOV3 parental cells were subjected to *ITGB3* knockdown. No difference in *TIMP1* expression was observed between these cells (Fig 5C and 5D), suggesting that the decreased level of *TIMP1* in PRKCZ-expressing cells was ITGB3-independent.

In addition to *ITGB3*, we examined if induction of IGF1 signalling has an effect on *TIMP1* expression. Upon IGF1 stimulation, SKOV3 parental and empty-vector controls exhibited a 2-fold decrease in *TIMP1* mRNA, but no further decrease in *TIMP1* expression was observed in PRKCZ clones (Fig 5E). This is an interesting finding, as IGF1 and PRKCZ signalling pathways may converge through their potential roles in the regulation of *TIMP1* expression.

### Effects of IGF and ITGB3 Signalling on Cell Migration in SKOV3 Cells

**Fig 6:**
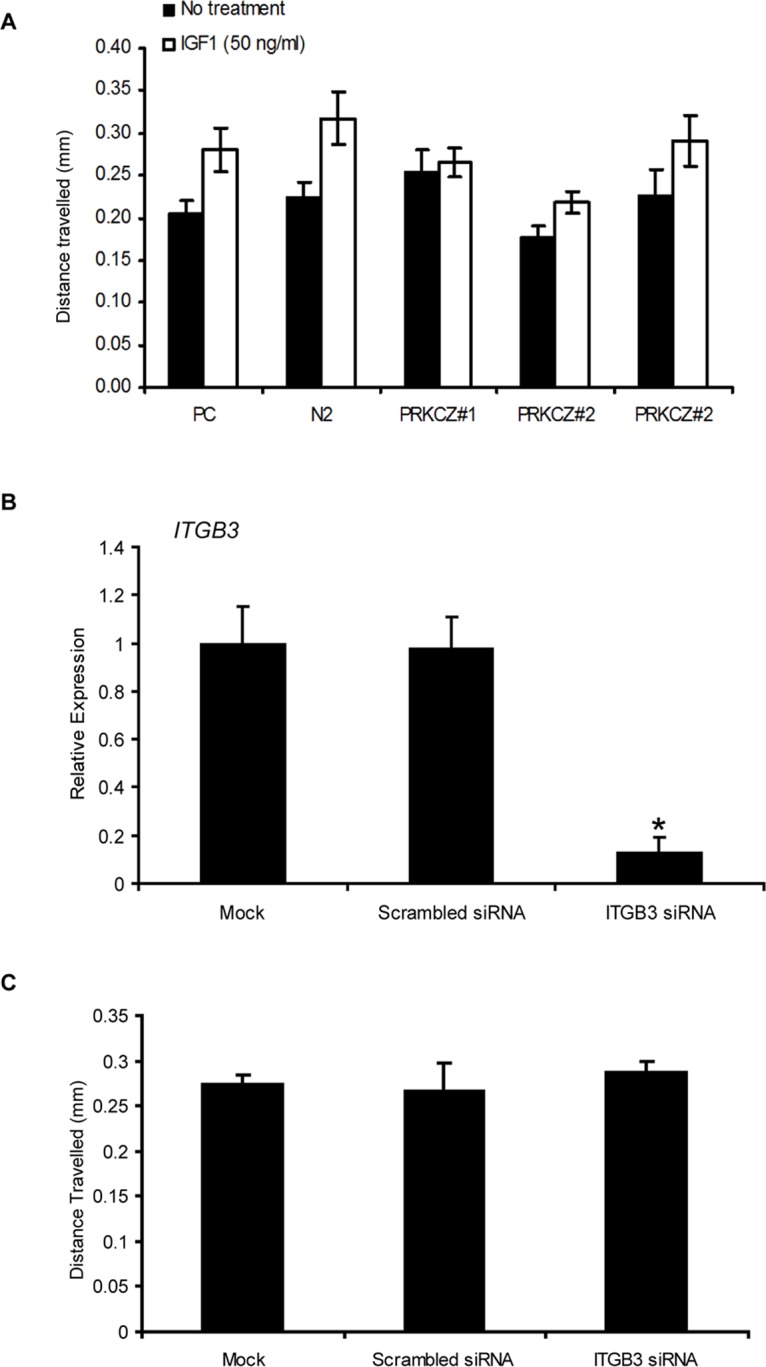
Effect of IGF1 and ITGB3 signalling on SKOV3 migration as observed by wound healing assay. (A) No significant increase in migration was observed between controls and PRKCZ-expressing SKOV3 cells in the absence of stimulation (black bars). However, upon treatment with IGF1, parental and vector-control cells showed an increase in migration (white bars; * p<0.05, n = 2). This observation was not seen in PRKCZ-expressing clones PC = parental control; N2 = empty vector control N2. (B, C) Knockdown of *ITGB3* has no effect on cell migration in SKOV3 cells. Confirmation of *ITGB3* knockdown in SKOV3 cells (*p<0.01), and distance travelled by SKOV3 cells with and without treatment of *ITGB3* siRNA (*p<0.05, n = 3).

The lack of migratory changes in PRKCZ-expressing SKOV3 cells as observed from the migration experiments as described above may be due to lack of stimulation. Since an increase in IGF1R expression was observed in cells over-expressing PRKCZ, similar migration assays were repeated with the addition of IGF1. An increase in migration was observed in parental and empty-vector controls upon stimulation with IGF1 in the wound healing assay (Fig 6A), illustrating that the IGF1 signalling pathway is involved in migration of SKOV3 cells. However, this effect was not observed in PRKCZ-expressing cells. The lack of response in PRKCZ-expressing cells may perhaps be due to negative feedback exerted by the over-expression of IGF1 receptor in these cells. Matrigel migration assays also did not show an increased level of invasion in any of cells upon stimulation of IGF1 (data not shown).

Lastly, to evaluate if ITGB3 plays a role in ovarian cancer migration, scratch wound healing assays were performed with SKOV3 cells treated with *ITGB3* siRNA. No difference in migration rate was observed between siRNA-treated and control cells (Fig 6B and 6C).

## Discussion

Human tumourigenesis is a multistep process in which cells can acquire properties, through genetic alterations, that allow them to transform to a higher malignant derivative [20]. In the present study, we aimed to determine if alteration in PRKCZ expression can drive such processes in ovarian cancer, including cell viability, proliferation, and cell migration.

PRKCZ has previously been implicated to be involved in various cancer cell types, including glioblastoma, prostate cancer, squamous cell carcinomas of the head and neck, squamous cervical cancer and soft tissue sarcomas [3,4,5,21,22,23,24,25]. Our current study revealed that PRKCZ also plays a role in proliferation in a subset of ovarian cancer cell lines, as it was observed that it affects cell viability in SKOV3 cells, but not OVCAR3 cells.

The ability of cancer cells to migrate during cancer progression is associated with the acquisition of abnormal motile behaviour resulting from various molecular alterations. Aberrant expression of PRKCZ is an example of such alteration, as its role in migration has previously been demonstrated in breast cancer, head and neck tumour cells, and pancreatic cancer [7,23,26,27,28]. Comparing migration of parental cell lines and PRKCZ-expressing counterparts indicated that over-expressing PRKCZ alone is not sufficient to exert increased migratory properties in the two ovarian cancer cell lines tested. Nevertheless, we demonstrated that siRNA knockdown of *PRKCZ* expression in SKOV3 parental cells can decrease its rate of migration. Our observation suggests that the endogenous level of PRKCZ is sufficient for cell motility in this particular ovarian cancer cell line.

In addition to PRKCZ, it is also important to note the potential roles of PRKCI (protein kinase C iota) in ovarian cancer. Similar to PRKCZ, PRKCI belongs to the atypical protein kinase C family group, and it has been implicated in the establishment of cell polarity, motility, proliferation, and survival of cancer cells [29,30,31,32]. Interestingly, the *PRKCI* gene has been shown to be amplified and over-expressed in serous epithelial ovarian cancers, and an increase in its DNA copy number is associated with a decrease in progression-free survival for the disease [30,33]. Since PRKCZ and PRKCI are highly homologous to one another, sharing ~70% overall amino acid sequence identity [34], it is possible that these two proteins function redundantly. Indeed, it has previously been demonstrated that disruption of either PRKCZ or PRKCI expression can inhibit tight junction formation in cultured epithelial cells, suggesting that these two proteins have an overlapping role in establishment of cell polarity [35]. For that reason, it may also be important to further investigate if PRKCI can contribute to the phenotype that we have observed in PRKCZ-expressing cells. Additionally, since siRNA knockdown of *PRKCZ* did not have an effect in OVCAR3 cells, it may be of interest to determine if these cell lines are more dependent on the activity of PRKCI.

PRKCZ is involved in various cell signalling pathways, thus may alter multiple downstream targets. To this end, we sought to identify some relevant molecular players that may be affected by PRKCZ expression.

A large body of evidence has supported the importance of IGF1R expression in ovarian cancer, and that increased expression of IGF1R is associated with aggressiveness, as well as drug resistance of the disease [15,16,36,37,38,39,40]. Our data indicate that over-expressing PRKCZ in certain ovarian cancer cell lines can alter the expression of IGF1R, and the type of expression alteration is cell-line dependent. Specifically, while no *IGF1R* transcript level alterations were observed in SKOV3 cells, the level of protein expression was found to be increased in cells that over-express PRKCZ, which may be explained by post-transcriptional processes such as protein translation, post-translation modification and decrease in protein degradation; however, the exact mechanism involved remains to be investigated. On the other hand, both gene and protein expression levels of IGF1R were found to be decreased in the OVCAR3 cell line when PRKCZ is over-expressed, suggesting that regulation of *IGF1R* by PRKCZ in this particular cell line may be occurring at the transcript level. These results suggest that regulation of IGF1R by PRKCZ may also be occurring within different biological pathways and may be dependent on other molecular characteristics specific to each of the cell lines, once again illustrating the heterogeneity of this disease, and further investigations are required to determine the exact mechanisms responsible for the dual effect PRKCZ has on IGF1R expression.

The role of ITGB3 in ovarian cancer has been implicated in a study by Kaur et al. in which over-expression of ITGB3 in SKOV3ip1 cells (cell line generated from ascites developed in nu/nu mouse by administering an intraperitoneal injection of SKOV3 cells) was found to be associated with decreased invasion, protease expression, as well as colony formation [41]. These observations were consistent with their subsequent *in vivo* experiments, which showed that tumours expressing ITGB3 were less aggressive compared to those that do not express this protein [41]. Moreover, upon examination of ITGB3 expression in ovarian tissue of patients with invasive ovarian cancer, the same group found that patients with high ITGB3 expression had a significantly better prognosis, a finding that is consistent with other recent studies [17,18,41]. Interestingly, our assessment of ITGB3 expression in PRKCZ-expressing SKOV3 and OVCAR3 ovarian cancer cells showed that ITGB3 is down-regulated in the presence of PRKCZ, as its gene and protein expressions were both decreased compared to controls. Future IHC studies should reveal whether this correlation occurs in ovarian tumour specimens.

The concomitant altered expression of IGF1R and ITGB3 in PRKCZ-expressing cells led to the question of whether these genes are activated in the same signalling pathway. Indeed, *IGF1R* siRNA knockdown in SKOV3 cells revealed that *ITGB3* transcription may be dependent on expression of IGF1R; however, the effects of IGF1R on OVCAR3 cells may differ since its expression is decreased in PRKCZ-expressing cells. Nevertheless, results from this study suggest one possible mechanism by which ITGB3 expression may be altered, and as a consequence, a more aggressive phenotype of ovarian cancer cells is developed.

Given that PRKCZ expression correlates with the expression of both IGF1R and ITGB3, we further examined if alteration of these genes can affect the migration phenotype of ovarian cancer cells that over-express PRKCZ. Interestingly, scratch wound healing migration assays showed that upon IGF1 stimulation, SKOV3 parental control cells, but not PRKCZ-expressing cells, displayed an increase in cellular motility, which was contrary to what was expected. The lack of response in PRKCZ-expressing cells may perhaps be due to a negative feedback mechanism exerted by the over-expression of IGF1 receptor in these cells, thus hindering the cells’ ability to respond to IGF1 signalling. Additionally, unlike results obtained from scratch wound assays, IGF1 stimulation had no effects on any of these cells in matrigel migration assays, suggesting that while IGF1 signalling may be involved in cell migration, it may be insufficient for increasing the invasive properties of these cells, because the cells are incapable of breaking down the matrigel matrix to cross the barrier.

**Fig 7:**
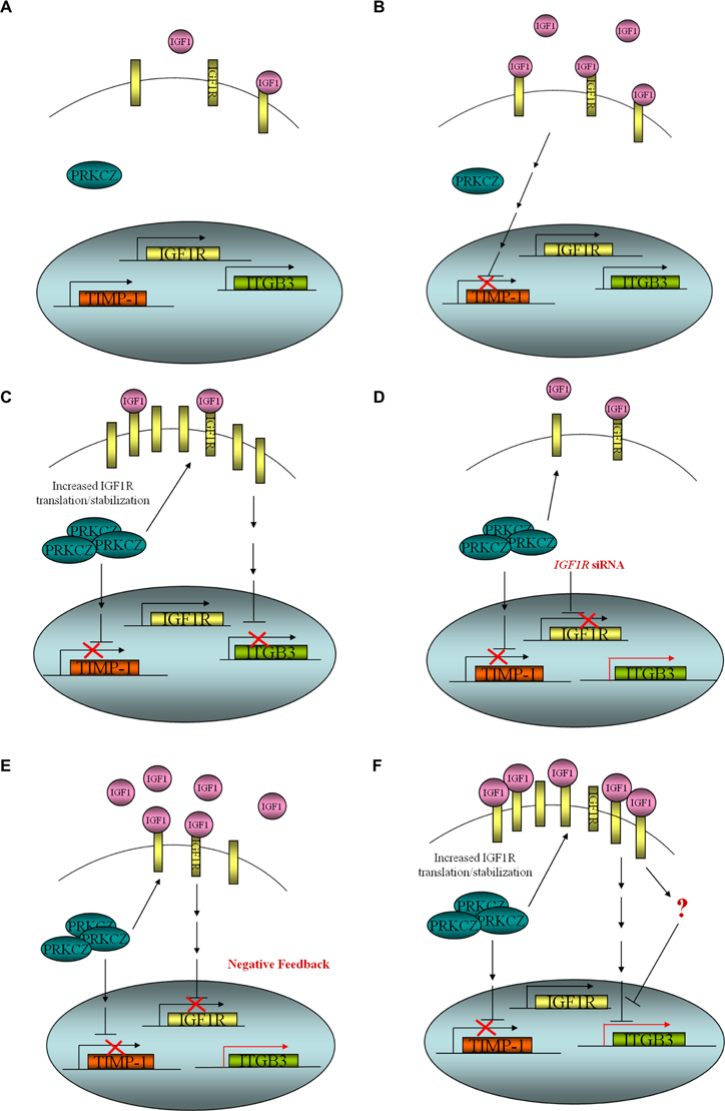
Proposed model of *ITGB3* and *TIMP1* transcriptional regulation through IGF-1 signalling in PRKCZ-expressing SKOV3 cells. (A) Expression of *IGF1R*, *ITGB3*, and *TIMP1* are in equilibrium in the presence of normal level of PRKCZ. (B) These same cells, when stimulated with IGF1, can decrease the expression of *TIMP1*. (C) Over-expression of PRKCZ increases either translation or stability of IGF1R, possibly leading to constitutive activation of IGF1 signalling cascade that results in transcriptional repression of *ITGB3* and increase cell survival through proliferation. (D) Repression of *ITGB3* in PRKCZ-expressing cells is dependent on IGF1R expression as *IGF1R* knockdown by siRNA derepresses its expression. (E) IGF1 stimulation activates negative feedback mechanism, leading to a decrease in *IGF1R* transcription; *ITGB3* expression is derepressed. (F) In addition to *IGF1R* transcription suppressor, other signalling pathway downstream of IGF1 cascade, such as PI3K/AKT, may be activated to derepress the expression of *ITGB3*.

Based on these observations, we propose the following model by which PRKCZ may participate during tumour progression in a subset of ovarian cancer (Fig 7). In cells with normal expression of PRKCZ, the expressions of *IGF1R*, *ITGB3*, and *TIMP1* are in equilibrium. These same cells, when stimulated with IGF1, can decrease the expression of *TIMP1*. When PRKCZ is deregulated and over-expressed, it increases the translation or stability of IGF1R, thus enhancing IGF1 signalling, leading to a repression of *ITGB3* expression, which ultimately can lead to changes in cellular processes that can enhance the aggressiveness of a tumour cell (eg. impaired apoptosis, increased proliferation). When *IGF1R* gene expression is decreased (eg. via siRNA knockdown) in these PRKCZ-expressing cells, rescue of *ITGB3* expression occurs. Additionally, when cells are stimulated with IGF1, the overall expression of IGF1R decreases due to a negative feedback mechanism that leads to suppression of *IGF1R* transcription and rescues the repression of *ITGB3*; however, there may be another yet to be identified pathway downstream of IGF1 signalling that can lead to derepression of *ITGB3*. One possible pathway may be PI3K/AKT, as IGF1 is a potent activator of this signalling cascade [42,43,44,45].

The results from our study suggest the potential roles of PRKCZ in ovarian cancer development; however, further investigations using an animal model are needed to elucidate the roles of PRKCZ and its molecular partners in ovarian cancer. A better understanding of the interaction of these molecules may be useful in development of therapeutics for the subset of ovarian cancer patients who display expression alterations of these genes.
